# Diethyl [(4-bromo­phen­yl)(5-chloro-2-hydroxy­anilino)meth­yl]phospho­nate

**DOI:** 10.1107/S1600536809043062

**Published:** 2009-10-28

**Authors:** V. H. H. Surendra Babu, M. Krishnaiah, G. Syam Prasad, C. Suresh Reddy, Rajni Kant

**Affiliations:** a Department of Physics, S.V. University, Tirupati 517 502, India; b Department of Chemistry, S.V. University, Tirupati 517 502, India; c Department of Physics, University of Jammu, Jammu Tawi 180 006, India

## Abstract

In the title compound, C_17_H_20_BrClNO_4_P, inter­molecular C—H⋯O and N—H⋯O hydrogen bonds form centrosymmetric *R*_2_^2^(10) dimers linked through O—H⋯O inter­molecular hydrogen bonds, which form centrosymmetric *R*_2_^2^(16) dimers. All these hydrogen bonds form chains along [010]. In addition, the crystal structure is stabilized by weak C—H⋯Br hydrogen bonds. The very weak intramolecular N—H⋯O interaction forms a five-membered ring.

## Related literature

For related structures, see: Krishnaiah *et al.* (2009 ▶); Yang *et al.* (2005 ▶).
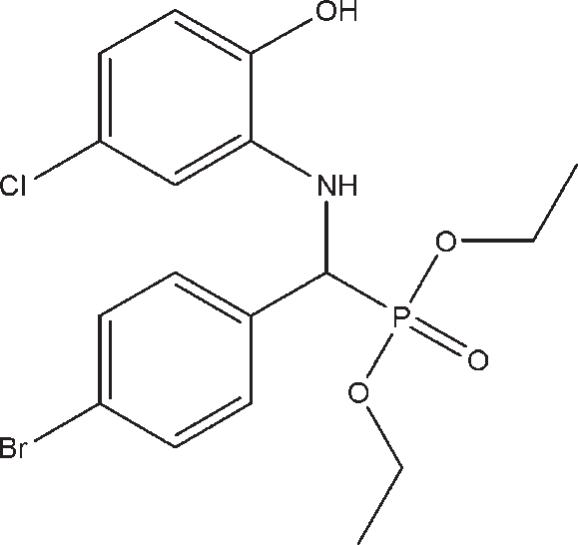


## Experimental

### 

#### Crystal data

C_17_H_20_BrClNO_4_P
*M* *_r_* = 448.66
Triclinic, 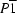

*a* = 7.8596 (15) Å
*b* = 9.1887 (13) Å
*c* = 14.425 (2) Å
α = 82.921 (13)°
β = 80.372 (15)°
γ = 70.701 (16)°
*V* = 966.8 (3) Å^3^
*Z* = 2
Mo *K*α radiation
μ = 2.37 mm^−1^
*T* = 293 K
0.30 × 0.24 × 0.18 mm

#### Data collection

Oxford Diffraction Xcalibur diffractometer
Absorption correction: multi-scan (*SADABS*; Sheldrick, 2004 ▶) *T*_min_ = 0.511, *T*_max_ = 0.653
12477 measured reflections
5846 independent reflections
2891 reflections with *I* > 2σ(*I*)
*R*_int_ = 0.031

#### Refinement

*R*[*F* ^2^ > 2σ(*F* ^2^)] = 0.056
*wR*(*F* ^2^) = 0.140
*S* = 1.05
5846 reflections
226 parameters
H-atom parameters constrained
Δρ_max_ = 0.60 e Å^−3^
Δρ_min_ = −0.54 e Å^−3^

### 

Data collection: *CryAlis Pro* (Oxford Diffraction, 2007 ▶); cell refinement: *CryAlis Pro*; data reduction: *CryAlis RED* (Oxford Diffraction, 2007 ▶); program(s) used to solve structure: *SHELXS86* (Sheldrick, 2008 ▶); program(s) used to refine structure: *SHELXL97* (Sheldrick, 2008 ▶); molecular graphics: *ZORTEPII* (Zsolnai, 1997 ▶); software used to prepare material for publication: *PARST* (Nardelli, 1995 ▶).

## Supplementary Material

Crystal structure: contains datablocks global, I. DOI: 10.1107/S1600536809043062/hg2579sup1.cif

Structure factors: contains datablocks I. DOI: 10.1107/S1600536809043062/hg2579Isup2.hkl

Additional supplementary materials:  crystallographic information; 3D view; checkCIF report

## Figures and Tables

**Table 1:** Hydrogen-bond geometry (Å, °)

*D*—H⋯*A*	*D*—H	H⋯*A*	*D*⋯*A*	*D*—H⋯*A*
N4—H4⋯O8^i^	0.86	2.47	3.287 (4)	159
C24—H24*A*⋯O5^ii^	0.97	2.53	3.472 (7)	163
O8—H8⋯O5^i^	0.82	1.90	2.615 (4)	145
C15—H15⋯Br2^iii^	0.98	2.99	3.945 (4)	164
N4—H4⋯O8	0.86	2.27	2.626 (4)	104

Symmetry codes: (i) 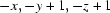; (ii) 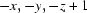; (iii) 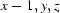.

**Fig. 1.**
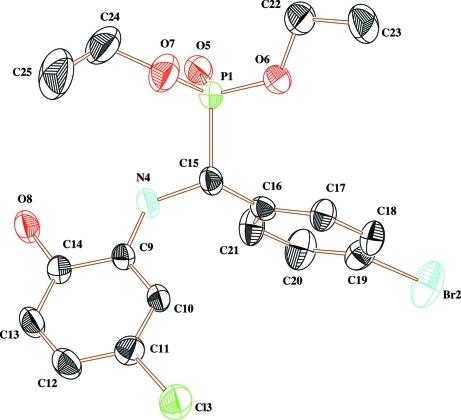
View of the molecule showing the atom-labelling scheme. Displacement ellipsoids are drawn at the 40% probability level.

**Fig. 2.**
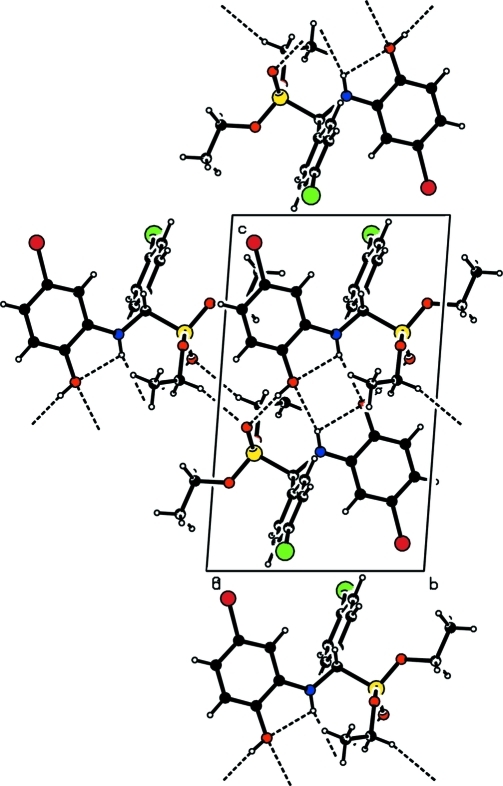
Packing of the molecule in the unit cell

## supplementary crystallographic information

### Comment

A similar co-ordination in hydrogen bonding, diethyl[(5-chloro- 2-hydroxyanilino)(4-chlorophenyl)methyl]phosphonate has been reported by us (Krishnaiah *et al.*, 2009). In continuation of our study on series of phosphonate compounds, we are now reporting the conformation of the structure of the title compound. The P=O(2) bond length is in good agreement with related structures (Krishnaiah *et al.*, 2009; Yang *et al.*, 2005). The bond angles O(2)—P(1)—O(1), O(2)—P(1)—O(3), O(2)—P(1)—C(7) are much larger than O(1)—P(1)—O(3) , O(1)—P(1)—C(7), O(3)—P(1)—C(7) bond angles, indicate a distorted tetrahedral around the phosphorus atom. The planar benzene rings are nearly perpendicular to each with dihedral angle of 78.1 (1)°. The P—O—C—C groups are in *trans* configuration avoiding steric interactions. The P(1)/O(1)/C(14)/C(15) group is nearly planar unlike the P(1)/O(3)/C(16)/C(17) group, the end atoms C(16)and C(17) are completely out of plane due to more thermal vibrations.

The O—H···O intermolecular hydrogen bonds act as a bridge between C—H···O intermolecular bonds, intra and intermolecular N—H···O hydrogen bonds. Here the phosphonate double bonded oxygen atom, which behaves as an acceptor participates in C—H···O intermolecular hydrogen bonding, whereas, the hydroxyl oxygen, which acts as both donor and acceptor, participates in the N—H···O intra and intermolecular hydrogen bonding. These hydrogen bond form chains along [010]. Additionally, the crystal structure is stabilized by C—H···Br hydrogen bonds.

### Experimental

To a stirred solution of 2-amino-4-chlorophenol (0.72 g, 0.005 mol), 4-bromo benzaldehyde (0.005 mol) in anhydrous toluene (15 ml) was added dropwise. Stirring was continued at room temperature of lh. Then diethylphosphite (0.7 g, 0.005 mol) in anhydrous toulene (15 ml) was added dropwise. Stirring was continued at room temperature for another 0.5 h, later the mixture was heated under reflux for 4–6 h. After completion of reaction (monitored by TLC) and the solvent was removed under reduced pressure. The resulting residue was purified by column chromatography on silica gel using petroleum ether-ethyl acetate (8:2) as eluent. Colorless, rectangular shaped single crystals were obtained for diffraction studies using methanol by slow evaporation.

### Refinement

H atoms bonded to N and O atoms were located in a difference map and refined with distance restraints of O—H = 0.82 and N—H = 0.86 Å, and with *U*_iso_(H) = 1.2*U*_eq_(N,*O*). Other H-atoms bound to carbon were positioned geometrically and refined using a riding model with d(C—H) = 0.93Å *U*_iso_=1.2_eq_ (C) for aromatic, C—H = 0.980Å *U*_iso_=1.2_eq_ (C) for methine, 0.97Å *U*_iso_ = 1.2_eq_ (C) for CH_2_ group and 0.96Å *U*_iso_ = 1.5_eq_ (C) for CH_3_ group.

### Crystal data

**Table:**

C_17_H_20_BrClNO_4_P	*Z* = 2
*M**_r_* = 448.66	*F*(000) = 456
Triclinic, *P*1	*D*_x_ = 1.541 Mg m^−^^3^
Hall symbol: -P 1	Mo *K*α radiation, λ = 0.71073 Å
*a* = 7.8596 (15) Å	Cell parameters from 5846 reflections
*b* = 9.1887 (13) Å	θ = 3.0–30.4°
*c* = 14.425 (2) Å	µ = 2.37 mm^−^^1^
α = 82.921 (13)°	*T* = 293 K
β = 80.372 (15)°	Rectangular, colorless
γ = 70.701 (16)°	0.30 × 0.24 × 0.18 mm
*V* = 966.8 (3) Å^3^	

### Data collection

**Table:**

Oxford Diffraction Xcalibur diffractometer	5846 independent reflections
Radiation source: fine-focus sealed tube	2891 reflections with *I* > 2σ(*I*)
graphite	*R*_int_ = 0.031
ω–2θ scans	θ_max_ = 30.4°, θ_min_ = 3.0°
Absorption correction: multi-scan (*SADABS*; Sheldrick, 2004)	*h* = −11→11
*T*_min_ = 0.511, *T*_max_ = 0.653	*k* = −13→12
12477 measured reflections	*l* = −20→20

### Refinement

**Table:**

Refinement on *F*^2^	Primary atom site location: structure-invariant direct methods
Least-squares matrix: full	Secondary atom site location: difference Fourier map
*R*[*F*^2^ > 2σ(*F*^2^)] = 0.056	Hydrogen site location: inferred from neighbouring sites
*wR*(*F*^2^) = 0.140	H-atom parameters constrained
*S* = 1.05	*w* = 1/[σ^2^(*F*_o_^2^) + (0.0511*P*)^2^ + 0.6969*P*] where *P* = (*F*_o_^2^ + 2*F*_c_^2^)/3
5846 reflections	(Δ/σ)_max_ < 0.001
226 parameters	Δρ_max_ = 0.60 e Å^−^^3^
0 restraints	Δρ_min_ = −0.54 e Å^−^^3^

### Special details

**Table:**

Geometry. All e.s.d.'s (except the e.s.d. in the dihedral angle between two l.s. planes) are estimated using the full covariance matrix. The cell e.s.d.'s are taken into account individually in the estimation of e.s.d.'s in distances, angles and torsion angles; correlations between e.s.d.'s in cell parameters are only used when they are defined by crystal symmetry. An approximate (isotropic) treatment of cell e.s.d.'s is used for estimating e.s.d.'s involving l.s. planes.
Refinement. Refinement of *F*^2^ against ALL reflections. The weighted *R*-factor *wR* and goodness of fit *S* are based on *F*^2^, conventional *R*-factors *R* are based on *F*, with *F* set to zero for negative *F*^2^. The threshold expression of *F*^2^ > σ(*F*^2^) is used only for calculating *R*-factors(gt) *etc*. and is not relevant to the choice of reflections for refinement. *R*-factors based on *F*^2^ are statistically about twice as large as those based on *F*, and *R*- factors based on ALL data will be even larger.Weighted least-squares planes through the starred atoms (Nardelli, Musatti, Domiano & Andreetti Ric.Sci.(1965),15(II—A),807). Equation of the plane: m1**X*+m2*Y+m3**Z*=dPlane 1 m1 = 0.92698(0.00062) m2 = 0.36804(0.00155) m3 = -0.07254(0.00162) D = 3.47552(0.01244) Atom d s d/s (d/s)**2 C1 * 0.0028 0.0034 0.823 0.678 C2 * 0.0007 0.0036 0.200 0.040 C3 * -0.0059 0.0040 - 1.479 2.188 C4 * 0.0072 0.0044 1.650 2.723 C5 * -0.0013 0.0041 - 0.319 0.102 C6 * -0.0029 0.0036 - 0.820 0.672 ============ Sum((d/s)**2) for starred atoms 6.403 Chi-squared at 95% for 3 degrees of freedom: 7.81 The group of atoms does not deviate significantly from planarityPlane 2 m1 = -0.16802(0.00159) m2 = 0.90248(0.00070) m3 = -0.39660(0.00142) D = 1.25137(0.01035) Atom d s d/s (d/s)**2 C8 * -0.0067 0.0030 - 2.243 5.033 C9 * 0.0049 0.0037 1.315 1.729 C10 * 0.0025 0.0039 0.638 0.407 C11 * -0.0045 0.0040 - 1.120 1.255 C12 * -0.0028 0.0048 - 0.589 0.347 C13 * 0.0112 0.0042 2.665 7.102 ============ Sum((d/s)**2) for starred atoms 15.874 Chi-squared at 95% for 3 degrees of freedom: 7.81 The group of atoms deviates significantly from planarityPlane 3 m1 = -0.36915(0.00155) m2 = 0.61070(0.00101) m3 = -0.70056(0.00061) D = -2.91653(0.00529) Atom d s d/s (d/s)**2 C15 * 0.0308 0.0053 5.844 34.149 C14 * -0.5236 0.0050 - 104.869 10997.448 O1 * 0.2184 0.0026 83.495 6971.443 P1 * -0.0205 0.0009 - 21.861 477.911 O3 * 0.2076 0.0031 66.126 4372.694 C16 * -0.5226 0.0056 - 93.878 8813.083 C17 * 0.3577 0.0071 50.270 2527.044 ============ Sum((d/s)**2) for starred atoms 34193.770 Chi-squared at 95% for 4 degrees of freedom: 9.49 The group of atoms deviates significantly from planarityPlane 4 m1 = -0.94870(0.00212) m2 = 0.26578(0.00425) m3 = -0.17128(0.00538) D = -2.37887(0.01364) Atom d s d/s (d/s)**2 P1 * 0.0007 0.0011 0.612 0.374 O1 * -0.0074 0.0032 - 2.332 5.440 C14 * -0.0127 0.0062 - 2.026 4.105 C15 * 0.0198 0.0063 3.175 10.079 ============ Sum((d/s)**2) for starred atoms 19.998 Chi-squared at 95% for 1 degrees of freedom: 3.84 The group of atoms deviates significantly from planarityPlane 5 m1 = 0.04035(0.00645) m2 = -0.93317(0.00349) m3 = 0.35715(0.00840) D = -0.00355(0.05973) Atom d s d/s (d/s)**2 P1 * -0.0057 0.0010 - 5.996 35.958 O3 * 0.0497 0.0032 15.570 242.410 C16 * 0.3855 0.0058 66.981 4486.437 C17 * -0.5407 0.0073 - 74.471 5545.978 ============ Sum((d/s)**2) for starred atoms 10310.782 Chi-squared at 95% for 1 degrees of freedom: 3.84 The group of atoms deviates significantly from planarityDihedral angles formed by LSQ-planes Plane - plane angle (s.u.) angle (s.u.) 1 2 78.16 (0.12) 101.84 (0.12) 1 3 86.18 (0.11) 93.82 (0.11) 1 4 39.72 (1/4) 140.28 (1/4) 1 5 70.61 (0.38) 109.39 (0.38) 2 3 27.00 (0.10) 153.00 (0.10) 2 4 62.15 (0.26) 117.85 (0.26) 2 5 7.86 (0.39) 172.14 (0.39) 3 4 50.76 (0.29) 129.24 (0.29) 3 5 33.39 (0.43) 146.61 (0.43) 4 5 69.67 (0.45) 110.33 (0.45)

### Fractional atomic coordinates and isotropic or equivalent isotropic displacement parameters (Å^2^)

**Table:**

	*x*	*y*	*z*	*U*_iso_*/*U*_eq_	
Br2	0.84116 (6)	0.35425 (7)	0.05533 (3)	0.0831 (2)	
P1	0.10786 (13)	0.17964 (11)	0.33103 (6)	0.0449 (2)	
Cl3	−0.27410 (17)	0.89126 (14)	0.07732 (7)	0.0780 (4)	
C14	−0.2049 (4)	0.7262 (4)	0.3792 (2)	0.0411 (7)	
C16	0.2724 (4)	0.3699 (3)	0.2163 (2)	0.0376 (7)	
N4	0.0040 (4)	0.4840 (3)	0.3349 (2)	0.0485 (7)	
H4	0.0234	0.4629	0.3927	0.058*	
O8	−0.1765 (4)	0.6665 (3)	0.46936 (17)	0.0567 (7)	
H8	−0.2379	0.7304	0.5067	0.085*	
C9	−0.1081 (4)	0.6286 (4)	0.3084 (2)	0.0382 (7)	
C15	0.0895 (5)	0.3672 (4)	0.2688 (2)	0.0423 (8)	
H15	0.0074	0.3829	0.2217	0.051*	
O5	0.2300 (4)	0.1371 (3)	0.40377 (17)	0.0572 (7)	
C11	−0.2492 (5)	0.8281 (4)	0.1956 (3)	0.0502 (9)	
O6	0.1667 (4)	0.0744 (3)	0.24775 (18)	0.0593 (7)	
C19	0.6080 (5)	0.3641 (5)	0.1206 (3)	0.0524 (9)	
C10	−0.1319 (4)	0.6816 (4)	0.2156 (2)	0.0419 (8)	
H10	−0.0691	0.6189	0.1669	0.050*	
O7	−0.0912 (4)	0.1837 (4)	0.3670 (2)	0.0753 (8)	
C17	0.3277 (5)	0.3198 (4)	0.1277 (2)	0.0487 (8)	
H17	0.2498	0.2873	0.0994	0.058*	
C13	−0.3194 (5)	0.8713 (4)	0.3567 (3)	0.0537 (9)	
H13	−0.3830	0.9349	0.4049	0.064*	
C18	0.4954 (5)	0.3157 (5)	0.0789 (2)	0.0538 (9)	
H18	0.5308	0.2806	0.0187	0.065*	
C20	0.5580 (6)	0.4167 (6)	0.2087 (3)	0.0685 (12)	
H20	0.6367	0.4491	0.2365	0.082*	
C12	−0.3424 (5)	0.9249 (4)	0.2649 (3)	0.0588 (10)	
H12	−0.4188	1.0238	0.2503	0.071*	
C21	0.3876 (5)	0.4210 (5)	0.2563 (3)	0.0581 (10)	
H21	0.3510	0.4587	0.3158	0.070*	
C22	0.1764 (8)	−0.0834 (5)	0.2531 (3)	0.0801 (14)	
H22A	0.0584	−0.0939	0.2786	0.096*	
H22B	0.2633	−0.1438	0.2948	0.096*	
C23	0.2321 (8)	−0.1402 (6)	0.1596 (4)	0.0888 (16)	
H23A	0.2394	−0.2471	0.1633	0.133*	
H23B	0.3491	−0.1299	0.1348	0.133*	
H23C	0.1447	−0.0812	0.1189	0.133*	
C24	−0.1716 (7)	0.1893 (7)	0.4638 (4)	0.0946 (17)	
H24A	−0.1842	0.0893	0.4873	0.114*	
H24B	−0.0929	0.2121	0.5011	0.114*	
C25	−0.3443 (8)	0.3037 (8)	0.4734 (5)	0.139 (3)	
H25A	−0.3946	0.3076	0.5388	0.208*	
H25B	−0.4236	0.2788	0.4386	0.208*	
H25C	−0.3319	0.4024	0.4495	0.208*	

### Atomic displacement parameters (Å^2^)

**Table:**

	*U*^11^	*U*^22^	*U*^33^	*U*^12^	*U*^13^	*U*^23^
Br2	0.0512 (3)	0.1413 (5)	0.0571 (3)	−0.0336 (3)	−0.00024 (19)	−0.0086 (3)
P1	0.0521 (5)	0.0414 (5)	0.0367 (5)	−0.0119 (4)	0.0040 (4)	−0.0078 (4)
Cl3	0.0924 (8)	0.0726 (7)	0.0466 (6)	0.0013 (6)	−0.0128 (5)	0.0068 (5)
C14	0.0396 (17)	0.0430 (19)	0.0380 (17)	−0.0091 (14)	−0.0025 (14)	−0.0076 (14)
C16	0.0396 (17)	0.0295 (16)	0.0382 (17)	−0.0026 (13)	−0.0092 (13)	−0.0004 (13)
N4	0.0571 (18)	0.0409 (16)	0.0337 (14)	0.0050 (13)	−0.0057 (13)	−0.0092 (12)
O8	0.0659 (16)	0.0500 (15)	0.0417 (14)	0.0012 (12)	−0.0063 (12)	−0.0128 (11)
C9	0.0331 (16)	0.0372 (18)	0.0404 (17)	−0.0076 (13)	0.0013 (13)	−0.0060 (14)
C15	0.0479 (19)	0.0409 (18)	0.0328 (16)	−0.0046 (15)	−0.0060 (14)	−0.0085 (14)
O5	0.0761 (18)	0.0456 (15)	0.0406 (14)	−0.0071 (13)	−0.0073 (12)	−0.0033 (11)
C11	0.046 (2)	0.053 (2)	0.047 (2)	−0.0094 (17)	−0.0110 (16)	0.0035 (17)
O6	0.0845 (19)	0.0450 (15)	0.0504 (15)	−0.0265 (13)	0.0051 (13)	−0.0142 (12)
C19	0.0420 (19)	0.065 (2)	0.048 (2)	−0.0172 (17)	−0.0069 (16)	0.0058 (18)
C10	0.0411 (18)	0.0381 (18)	0.0394 (18)	−0.0041 (14)	0.0005 (14)	−0.0077 (14)
O7	0.0709 (19)	0.092 (2)	0.0655 (19)	−0.0371 (17)	0.0143 (15)	−0.0151 (16)
C17	0.052 (2)	0.062 (2)	0.0355 (18)	−0.0195 (18)	−0.0062 (15)	−0.0102 (16)
C13	0.056 (2)	0.041 (2)	0.050 (2)	0.0055 (17)	−0.0049 (17)	−0.0109 (16)
C18	0.054 (2)	0.070 (3)	0.0354 (18)	−0.0170 (19)	0.0011 (16)	−0.0130 (17)
C20	0.061 (3)	0.100 (3)	0.054 (2)	−0.033 (2)	−0.007 (2)	−0.019 (2)
C12	0.056 (2)	0.042 (2)	0.065 (3)	0.0038 (17)	−0.0085 (19)	−0.0071 (18)
C21	0.056 (2)	0.079 (3)	0.043 (2)	−0.022 (2)	−0.0002 (17)	−0.0222 (19)
C22	0.115 (4)	0.065 (3)	0.068 (3)	−0.048 (3)	0.019 (3)	−0.023 (2)
C23	0.113 (4)	0.074 (3)	0.086 (4)	−0.040 (3)	0.018 (3)	−0.042 (3)
C24	0.077 (3)	0.105 (4)	0.080 (3)	−0.023 (3)	0.021 (3)	0.015 (3)
C25	0.086 (4)	0.154 (6)	0.115 (5)	0.015 (4)	0.039 (4)	0.002 (4)

### Geometric parameters (Å, °)

**Table:**

Br2—C19	1.894 (4)	C10—H10	0.9300
P1—O5	1.471 (3)	O7—C24	1.433 (6)
P1—O6	1.548 (3)	C17—C18	1.378 (5)
P1—O7	1.553 (3)	C17—H17	0.9300
P1—C15	1.815 (3)	C13—C12	1.375 (5)
Cl3—C11	1.755 (4)	C13—H13	0.9300
C14—O8	1.372 (4)	C18—H18	0.9300
C14—C13	1.375 (5)	C20—C21	1.389 (6)
C14—C9	1.394 (4)	C20—H20	0.9300
C16—C17	1.364 (5)	C12—H12	0.9300
C16—C21	1.377 (5)	C21—H21	0.9300
C16—C15	1.515 (5)	C22—C23	1.452 (6)
N4—C9	1.379 (4)	C22—H22A	0.9700
N4—C15	1.440 (4)	C22—H22B	0.9700
N4—H4	0.8600	C23—H23A	0.9600
O8—H8	0.8200	C23—H23B	0.9600
C9—C10	1.388 (4)	C23—H23C	0.9600
C15—H15	0.9800	C24—C25	1.413 (7)
C11—C12	1.367 (5)	C24—H24A	0.9700
C11—C10	1.385 (5)	C24—H24B	0.9700
O6—C22	1.420 (5)	C25—H25A	0.9600
C19—C18	1.358 (5)	C25—H25B	0.9600
C19—C20	1.365 (6)	C25—H25C	0.9600
			
O5—P1—O6	115.61 (15)	C12—C13—C14	121.8 (3)
O5—P1—O7	115.13 (17)	C12—C13—H13	119.1
O6—P1—O7	104.18 (16)	C14—C13—H13	119.1
O5—P1—C15	114.15 (16)	C19—C18—C17	118.7 (3)
O6—P1—C15	100.95 (15)	C19—C18—H18	120.7
O7—P1—C15	105.21 (17)	C17—C18—H18	120.7
O8—C14—C13	124.4 (3)	C19—C20—C21	118.9 (4)
O8—C14—C9	115.3 (3)	C19—C20—H20	120.6
C13—C14—C9	120.3 (3)	C21—C20—H20	120.6
C17—C16—C21	118.2 (3)	C11—C12—C13	117.8 (3)
C17—C16—C15	120.3 (3)	C11—C12—H12	121.1
C21—C16—C15	121.4 (3)	C13—C12—H12	121.1
C9—N4—C15	122.0 (3)	C16—C21—C20	120.7 (3)
C9—N4—H4	119.0	C16—C21—H21	119.6
C15—N4—H4	119.0	C20—C21—H21	119.6
C14—O8—H8	109.5	O6—C22—C23	109.5 (4)
N4—C9—C10	123.9 (3)	O6—C22—H22A	109.8
N4—C9—C14	117.9 (3)	C23—C22—H22A	109.8
C10—C9—C14	118.2 (3)	O6—C22—H22B	109.8
N4—C15—C16	115.6 (3)	C23—C22—H22B	109.8
N4—C15—P1	108.2 (2)	H22A—C22—H22B	108.2
C16—C15—P1	110.7 (2)	C22—C23—H23A	109.5
N4—C15—H15	107.4	C22—C23—H23B	109.5
C16—C15—H15	107.4	H23A—C23—H23B	109.5
P1—C15—H15	107.4	C22—C23—H23C	109.5
C12—C11—C10	122.0 (3)	H23A—C23—H23C	109.5
C12—C11—Cl3	119.5 (3)	H23B—C23—H23C	109.5
C10—C11—Cl3	118.5 (3)	C25—C24—O7	110.6 (5)
C22—O6—P1	126.1 (3)	C25—C24—H24A	109.5
C18—C19—C20	121.5 (4)	O7—C24—H24A	109.5
C18—C19—Br2	119.0 (3)	C25—C24—H24B	109.5
C20—C19—Br2	119.6 (3)	O7—C24—H24B	109.5
C11—C10—C9	119.9 (3)	H24A—C24—H24B	108.1
C11—C10—H10	120.0	C24—C25—H25A	109.5
C9—C10—H10	120.0	C24—C25—H25B	109.5
C24—O7—P1	124.8 (3)	H25A—C25—H25B	109.5
C16—C17—C18	122.0 (3)	C24—C25—H25C	109.5
C16—C17—H17	119.0	H25A—C25—H25C	109.5
C18—C17—H17	119.0	H25B—C25—H25C	109.5
			
C15—N4—C9—C10	−8.0 (5)	N4—C9—C10—C11	179.2 (3)
C15—N4—C9—C14	171.1 (3)	C14—C9—C10—C11	0.1 (5)
O8—C14—C9—N4	0.3 (4)	O5—P1—O7—C24	16.9 (4)
C13—C14—C9—N4	−179.6 (3)	O6—P1—O7—C24	144.5 (4)
O8—C14—C9—C10	179.5 (3)	C15—P1—O7—C24	−109.7 (4)
C13—C14—C9—C10	−0.4 (5)	C21—C16—C17—C18	1.5 (5)
C9—N4—C15—C16	88.1 (4)	C15—C16—C17—C18	−177.9 (3)
C9—N4—C15—P1	−147.2 (3)	O8—C14—C13—C12	−180.0 (4)
C17—C16—C15—N4	−150.5 (3)	C9—C14—C13—C12	−0.1 (6)
C21—C16—C15—N4	30.1 (4)	C20—C19—C18—C17	−0.3 (6)
C17—C16—C15—P1	86.1 (3)	Br2—C19—C18—C17	178.5 (3)
C21—C16—C15—P1	−93.3 (3)	C16—C17—C18—C19	−0.4 (6)
O5—P1—C15—N4	−65.4 (3)	C18—C19—C20—C21	−0.2 (7)
O6—P1—C15—N4	169.9 (2)	Br2—C19—C20—C21	−179.0 (3)
O7—P1—C15—N4	61.7 (3)	C10—C11—C12—C13	−1.4 (6)
O5—P1—C15—C16	62.1 (3)	Cl3—C11—C12—C13	180.0 (3)
O6—P1—C15—C16	−62.6 (3)	C14—C13—C12—C11	0.9 (6)
O7—P1—C15—C16	−170.7 (2)	C17—C16—C21—C20	−2.1 (6)
O5—P1—O6—C22	66.1 (4)	C15—C16—C21—C20	177.3 (4)
O7—P1—O6—C22	−61.3 (4)	C19—C20—C21—C16	1.4 (7)
C15—P1—O6—C22	−170.2 (4)	P1—O6—C22—C23	178.3 (3)
C12—C11—C10—C9	0.9 (6)	P1—O7—C24—C25	132.8 (5)
Cl3—C11—C10—C9	179.5 (3)		

### Hydrogen-bond geometry (Å, °)

**Table:**

*D*—H···*A*	*D*—H	H···*A*	*D*···*A*	*D*—H···*A*
N4—H4···O8^i^	0.86	2.47	3.287 (4)	159
C24—H24A···O5^ii^	0.97	2.53	3.472 (7)	163
O8—H8···O5^i^	0.82	1.90	2.615 (4)	145
C15—H15···Br2^iii^	0.98	2.99	3.945 (4)	164
N4—H4···O8	0.86	2.27	2.626 (4)	104

Symmetry codes: (i) −*x*, −*y*+1, −*z*+1; (ii) −*x*, −*y*, −*z*+1; (iii) *x*−1, *y*, *z*.
